# Reframing sedation for existential suffering: A case for integrating rapid-relief strategies

**DOI:** 10.1017/S1478951525101612

**Published:** 2026-01-13

**Authors:** Juan Esteban Correa-Morales, Nidia Mantilla-Manoslava, Joaquim Julià-Torras

**Affiliations:** 1 Faculty of Medicine and Health Sciences, UIC Barcelona – Universitat Internacional de Catalunya, Sant Cugat del Vallès, Barcelona, Spain; 2 Palliative care department, Hospital Internacional de Colombia, Bucaramanga, Colombia; 3 Palliative Care Department, Institut Català d’Oncologia, Badalona, Barcelona, España

A patient has acute pain. The physician decides to bypass exploration and initiates gabapentin. The next day, pain persists. The physician, despite knowing the medication’s delayed onset, declares the pain refractory and proceeds to sedate the patient. Even though this example is deliberately stemmed, most clinicians would consider such a decision unacceptable in the context of pain.

According to the revised European Association of Palliative Care (EAPC) guidelines on palliative sedation, a symptom is considered refractory when, after appropriate assessment and standard treatment, no further interventions are expected to provide adequate relief within an acceptable time frame or without an unacceptable risk–benefit ratio for the patient (Surges et al. 2024). Within the revised EAPC framework, a symptom is deemed “untreatable by healthcare professionals” when, after a thorough and expert clinical evaluation, no additional interventions are expected to achieve adequate relief within a timeframe compatible with the patient’s condition, or when the remaining treatment options would entail an unacceptable burden or risk (Morita et al. 2002). Intractability goes beyond the mere absence of available therapies: it reflects a professional judgement that further measures are unlikely to be effective, timely, or tolerable. In the case of existential suffering (where psychotherapeutic, psychosocial, and spiritual interventions may still offer meaningful benefit) establishing intractability is particularly challenging and requires interdisciplinary assessment, as the threshold for declaring refractoriness is considerably higher than for physical symptoms (Boston et al. 2011; Thomas et al. 2024).

A closer examination of the “*unbearable*” component of the definition is warranted. Existential suffering is deeply subjective and multidimensional, making clinical assessment inherently difficult (Boston et al. 2011). Nevertheless, validated instruments exist that serve as proxies to systematically assess key dimensions of existential suffering. The Patient Dignity Inventory (Chochinov et al. 2008) evaluates dignity-related psychological and existential concerns; the Demoralization Scale (and its revised and short versions) (Robinson et al. 2016) quantifies demoralization and tools such as the Desire for Death Rating Scale (Chochinov et al. 1995), the Schedule of Attitudes toward Hastened Death (including its short SAHD-10 version) (Kremeike et al. 2025), and the AFEDD (Assessment Frequency and Extent Desire to Death) (Rodríguez-Prat et al. 2017) facilitates structured clinical interview for the assessment of the wish to hasten death. These proxy measures capture key indicators of existential suffering that require optimal management through coordinated interdisciplinary palliative care, rather than isolated clinical decision-making (Ferrell and Paice 2019).

That brings us to one of the two current theory-practice gaps related to using sedation for existential suffering: even if the patient experiences unbearable suffering, it is not necessarily untreatable (and therefore not truly refractory). This is because the assessment of treatment benefit is often made prematurely, either before therapy is initiated or before its benefit has had a chance to manifest. Due to this, in clinical practice, relief sedation is used for not yet refractory existential suffering, but as a bridge therapy for immediate relief while waiting for long-term treatments onset. This distinction is not contemplated in any guideline and is of utmost importance nowadays, as some countries have established laws permitting patients to request euthanasia or medical aid in dying when their suffering is categorized as unbearable due to its refractoriness. It would also help physicians to better understand the symptom course and therefore guide their practice about the level of sedation provided (Rodrigues et al. 2018; Gabl et al. 2024; Maeda et al. 2025).

The second theory-practice gap arises from deeming existential suffering refractory and administering sedation (even as a bridge therapy) when readily available short-onset treatments are not utilized. Growing evidence points to the potential of rapid-onset therapies for existential suffering, such as psilocybin (Griffiths et al. 2016; Borgogna et al. 2025; Alexander et al. 2025), ketamine (Decazes et al. 2023; Sholevar et al. 2025), dexmedetomidine (Gaertner and Fusi-Schmidhauser 2022; Tate and Ferguson 2025), and magnetic stimulation (Watt et al. 2022; Downar et al. 2024; Kaster et al. 2024). All these interventions are supported by randomized controlled trials in research conducted outside the palliative care setting and the evidence specifically within palliative care remains limited. Despite this emerging evidence, these treatments have not yet been systematically incorporated into routine clinical practice, reflecting a persistent disconnect between available therapeutic options and their real-world implementation.

We can think of four possible explanations for this situation. The first one is that these treatments lack sufficient evidence to recommend their use as first-line therapy: this perspective is not entirely accurate as while their efficacy remains under scrutiny, there are systematic reviews supporting their safety profile. In stark contrast, sedating a patient for an unspecified duration due to existential suffering, without considering established life expectancy criteria, is a practice supported by significantly less evidence. If this situation is considered properly as what it is, a medical emergency, it is more rational to utilize safe treatments that, even if not fully effective, may offer helpful, rapid relief and serve as a bridge to psychotherapy. The second explanation is that these treatments are unavailable in most places, which precludes their wide recommendation. However, if international guidelines fail to recommend them as first-line therapy this hinders adoption and perpetuates their availability. A third argument suggests that psychotherapeutic options are superior and should therefore constitute the first line of treatment, despite their slower onset. Current data show scarce head-to-head evidence to definitively assert the superiority of psychotherapy over rapid-onset interventions (Lee 2020; Wong and Yu 2021; Terao and Satoh 2022). Consequently, these treatments are best viewed as sequential and complementary. This approach aligns with standard medical practice. Examples illustrating this principle in physical symptoms include the temporary employment of a rapidly acting opioid like morphine as a rescue medication for pain while an agent such as methadone is titrated. The last explanation could be related to avoiding medicalization of suffering. This concern is eased by the fact that short-onset therapies often include psychotherapeutic elements, unlike sedation, which is itself a form of medicalization.

Current guidelines addressing spirituality, the wish to hasten death, and the management of existential suffering require updating to incorporate the emerging evidence on rapid-onset interventions. The lack of guidance fuels theory–practice gaps, leaving clinicians with rigid, often suboptimal options and no middle-ground pathway. Despite valid concerns, the integration of rapid-onset interventions calls for a unified framework that bridges pharmacologic and psychotherapeutic care. Incorporating this stepwise, multimodal perspective would help narrow existing theory–practice discrepancies and better support clinicians in responding to existential suffering before it progresses to a truly refractory state.
